# Advancing Postoperative Pain Management in Oral Cancer Patients: A Systematic Review

**DOI:** 10.3390/ph17040542

**Published:** 2024-04-22

**Authors:** Angelo Michele Inchingolo, Gianna Dipalma, Alessio Danilo Inchingolo, Irene Palumbo, Mariafrancesca Guglielmo, Roberta Morolla, Antonio Mancini, Francesco Inchingolo

**Affiliations:** Department of Interdisciplinary Medicine, School of Medicine, University of Bari “Aldo Moro”, 70124 Bari, Italy; angeloinchingolo@gmail.com (A.M.I.); ad.inchingolo@libero.it (A.D.I.); irenepalu@icloud.com (I.P.); m.guglielmo2@studenti.uniba.it (M.G.); robertamorolla@gmail.com (R.M.); dr.antonio.mancini@gmail.com (A.M.)

**Keywords:** postoperative pain, cancer-related pain, oral cancer, head and neck cancer, oral squamous cell carcinoma, postoperative analgesia, opioid

## Abstract

The goal of this review is to shed light on the management of orofacial discomfort after a cancer diagnosis in the head and neck region. A search was conducted on PubMed, Scopus, and Web of Science to identify studies on postoperative pain control in oral cancer. The review included open-access research, investigations into pain management, randomized clinical trials, retrospective studies, case-control studies, prospective studies, English-written studies, and full-text publications. Exclusion criteria included animal studies; in vitro studies; off-topic studies; reviews, case reports, letters, or comments; and non-English language. Three reviewers independently accessed databases and assigned a quality rating to the chosen articles. The review explores postoperative pain management in oral cancer patients; highlighting persistent opioid use; the efficacy of adjuvant drugs, such as gabapentin; and a multimodal approach. It emphasizes the need for personalized pain management, recognizing individual pain perception and tailoring interventions. Integrating pharmacological and non-pharmacological strategies is crucial for comprehensive pain management. The review also serves as a guide for future research, emphasizing the need for standardized methodologies and diverse participant populations.

## 1. Introduction

Pain is characterized as “a disagreeable sensory or emotional encounter linked with real or potential damage to tissue, or articulated about such damage” [1,2]. The severity of pain frequently does not correlate with the nature or degree of tissue injury [3]. The perception of pain is individual and is a multifaceted phenomenon that encompasses psychological and emotional processes along with nociceptive and non-nociceptive impulses in ascending pathways [4,5,6]. These pathways are, in turn, connected to the activation of descending pain-inhibitory systems [2,7,8].

Cancer-related pain may arise from tumor progression, invasive diagnostic or therapeutic procedures, chemotherapy and radiotherapy toxicity, infections, or muscular pains [9,10,11,12,13]. The general belief is that cancer pain can be effectively managed in around 90 out of 100 patients [14,15]. Despite this, undertreatment is prevalent, likely stemming from factors such as negative attitudes towards the use of certain pain relief drugs and a lack of knowledge among physicians regarding effective management [16,17,18,19,20,21,22].

Numerous challenges contribute to the complexity of managing pain in head and neck cancer: the extensive innervation of the head and neck, the development of pain resulting from chemotherapy and radiotherapy, the invasive characteristics of tumors in this area, and the occurrence of pain triggered by functional movements (such as chewing, swallowing, or talking) [23,24,25,26,27,28,29].

### 1.1. Prevalence

In cases of oral cavity tumors, pain may be the symptom that precedes diagnosis. In fact, in this regard, more than 60% of patients with cancer in the head and neck region report discomfort or actual pain in the first 6 months [30,31,32,33,34,35,36,37]. 

In patients with oral squamous cell carcinoma (SCC), the most often reported symptom is oral discomfort and, later, also pain [38,39,40,41,42]. The latter results frequently occur in the anterior portion of the tongue, while lesions located at the base of the tongue mainly cause odynophagia, otalgia, and dysphagia [43,44,45,46]. These symptoms have been recorded with a higher prevalence in men than in women [47,48,49,50,51,52,53].

### 1.2. Management of Oral Cancer Pain

The World Health Organization (WHO) has advocated for the utilization of a rigorously validated methodology, referred to as the WHO scale, to address pain according to its intensity [54,55]. This protocol is grounded in five fundamental principles for the pharmacological management of cancer-related pain: by the mouth, by the clock, by the ladder, for the individual, and with attention to detail [56,57]. 

The initial approach involves the utilization of non-steroidal anti-inflammatory drugs (NSAIDs) e.g., naproxen, diclofenac, or indomethacin, for managing mild-to-moderate pain [58,59]. If the pain persists, an opioid (such as codeine or hydrocodone) should be introduced in conjunction with the NSAID [60,61,62,63,64,65]. For persistent or initially moderate-to-severe pain, an escalation in opioid potency (mainly morphine, methadone, or fentanyl) is recommended [66,67,68]. In cases where oral administration is not feasible, an exploration of rectal or transdermal routes is advised, reserving parenteral routes (such as subcutaneous or intravenous) for situations where simpler methods prove ineffective [69,70,71].

**Figure 1:**
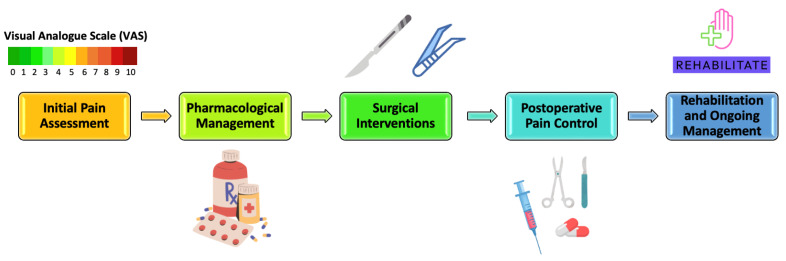
Pain Management Spectrum for Oral Cancer Patients.

Surgical interventions prove beneficial for specific patients by reducing tumor size through debulking, consequently alleviating symptoms associated with obstruction or compression [72,73,74]. Pain management typically takes a secondary role during curative tumor resection, while it commonly becomes the primary objective in palliative surgery for unresectable tumors (Figure 1) [75,76,77]. 

### 1.3. Management of Oral Cancer Pain after Therapy

Surgical interventions can result in substantial alterations in anatomy and functionality for patients, necessitating subsequent rehabilitation and ongoing pain management [78,79]. Employing precise surgical techniques can mitigate the severity of postoperative pain [80,81]. The careful handling of tissues, utilization of nerve- and vessel-sparing procedures, avoidance of tissue ischemia, and selection of non-muscle-splitting incisions collectively contribute to minimizing surgical pain and facilitating recovery [82,83]. Immediately after the operation, a comprehensive pain-control approach may be applied [84,85].

While numerous adverse effects of cancer treatment are now well managed, certain outcomes, such as mucositis and salivary gland hypofunction, remain nearly inevitable consequences of oral cancer treatment [86,87,88]. Mucositis, particularly during chemotherapy and radiation, is a debilitating and painful condition with potential interruptions and dose reductions impacting treatment outcomes [81,89,90,91,92,93,94,95]. Pain intensity in mucositis is linked to tissue damage and local inflammation, requiring aggressive analgesic management, often involving opioids [96]. There is no conclusive evidence favoring patient-controlled analgesia over continuous infusion, but the former results in reduced opioid usage per hour and shorter pain duration [97,98].

Laser treatment is also thought to be beneficial in avoiding and relieving mouth discomfort caused by radiation therapy and chemotherapy [99,100,101].

Although the literature on the oral cancer’s management is scarce even today, the purpose of this review is precisely to shed light on the management of orofacial pain following cancer onset in the head and neck region.

## 2. Materials and Methods

### 2.1. Protocol and Registration

The protocol for this systematic review was registered at PROSPERO with the ID: CRD 501771, and it was carried out in accordance with Preferred Reporting Items for Systematic Reviews and Meta-Analyses (PRISMA) [102].

### 2.2. Search Processing

To identify studies on the subject of postoperative pain control in oral cancer, a search was conducted on PubMed, Scopus, and Web of Science for publications published between 1 January 2003 and 31 December 2023. Boolean keywords were employed in the search strategy: (oral cancer) AND ((postoperative pain) OR (postoperative analgesia)) (Table 1).

### 2.3. Inclusion Criteria

The subsequent inclusion criteria were taken into consideration: (1) research with open access; (2) investigations into the management of pain following surgery in cases of oral cancer; (3) randomized clinical trials, retrospective studies, case-control studies, and prospective studies; (4) studies written in English; and (5) full-text publications.

Papers that did not meet the specified requirements were not accepted. 

The review was conducted using the PICOS criteria:

- Participants: adults, both male and female;
- Interventions: pain control in the oral cancer;
- Comparisons: different drugs utilized;
- Outcomes: the review underscores diverse drug interventions in managing oral cancer pain, emphasizing the need for nuanced, patient-specific approaches and calling for more in-depth research in the field.
- Study: randomized clinical trials, retrospective studies, case-control studies, and prospective studies.

### 2.4. Exclusion Criteria

The exclusion criteria were as follows: (1) animal studies; (2) in vitro studies; (3) off-topic; (4) reviews, case reports, case series, letters, or comments; (5) non-English language.

### 2.5. Data Processing

Based on selection criteria, three reviewers (M.G., I.P., and R.M.) independently accessed the databases to gather the studies and assigned a quality rating. Zotero (version 6.0.15) was loaded with the chosen articles. Disagreements amongst the three writers were resolved through consultation with a senior reviewer (F.I.).

## 3. Results

### Study Selection and Characteristics

A total of 2933 publications were found using the electronic database search (Scopus *n* = 1656, PubMed *n* = 656, Web of Science *n* = 621), but no articles were found using the manual search.

**Figure 2:**
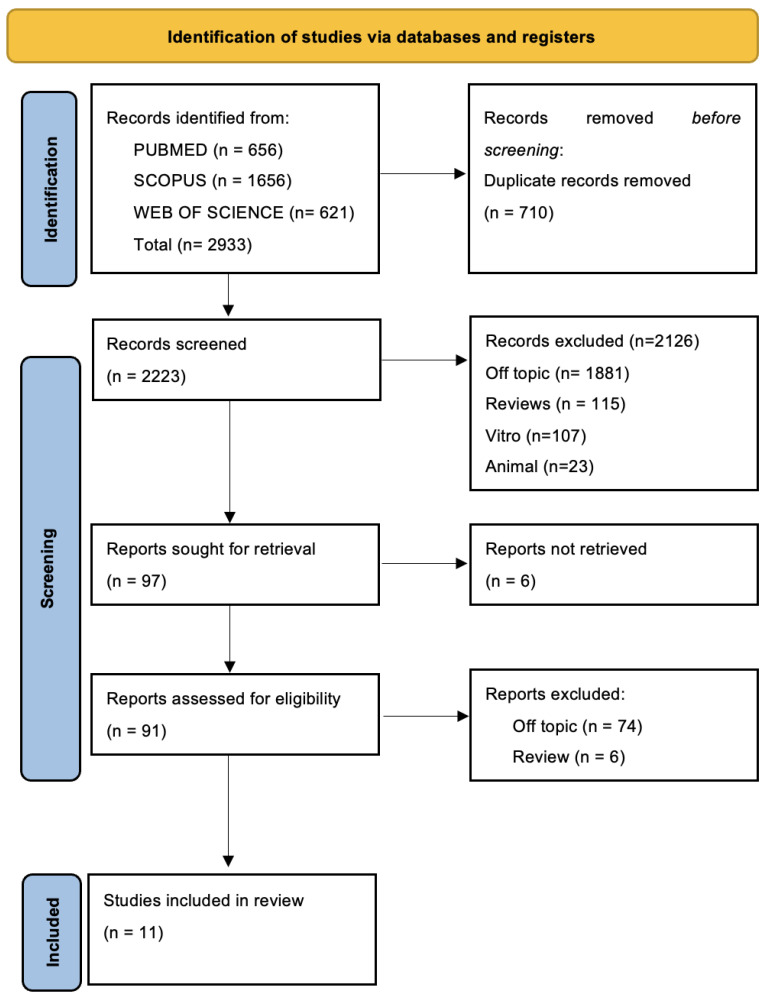
PRISMA flow diagram and database search indicators.

Following the removal of duplicates (*n* = 710), the titles and abstracts of 2223 studies were assessed in order to filter them. An amount of 97 records were chosen out of 2126 papers that did not match the inclusion criteria (1881 off-topic, 115 reviews, 107 vitro experiments, 23 animal research). Six records that could not be retrieved were subsequently eliminated, and ninety-one reports that did not fit the inclusion requirements were eliminated as well (seventy-four off-topic, six reviews). Eleven records were chosen for a qualitative analysis after being deemed eligible. Figure 2 and Table 2, respectively, present the selection procedure and the summary of the records that were chosen.

## 4. Discussion

Managing pain in individuals with oral cancer poses a significant challenge for healthcare practitioners, necessitating a thorough and individualized approach. The scientific literature offers a comprehensive and varied overview of methods, tactics, and treatments aimed at enhancing postoperative pain management and enhancing the overall well-being of patients undergoing surgical procedures. A review of the literature allows for an in-depth exploration of different perspectives presented in research studies, investigating crucial aspects that define the approach to pain control in patients with oral cancer.

### 4.1. Persistent Opioid Use and Pre-Operative Factors

A critical point underscored in the literature is the continued use of opioids during the postoperative phase. Cata et al.’s study, focusing on pain management in patients undergoing surgery for oral tongue tumors from January 2004 to January 2018, reveals that 15% of patients continued to use opioids one year after the surgery. This underscores the importance of implementing perioperative measures to effectively address pain in this specific patient cohort. Furthermore, Cata et al.’s study identified significant correlations between adjuvant therapies, preoperative opioid use, and preoperative pain with chronic and persistent postoperative opioid use [103]. In contrast to other malignancies, patients with oral tongue cancer demonstrated a higher likelihood of chronic postoperative opioid use, emphasizing the need to consider variables such as pain severity and opioid use before surgery when predicting long-term opioid use.

Another intriguing approach emerging from the literature is the use of dexmedetomidine in postoperative pain management. Gupta et al.’s study compared the efficacy of dexmedetomidine and fentanyl in oral cancer surgery patients [107]. The results showed better hemodynamic stability, reduced postoperative pain, and lower analgesic requirements in the dexmedetomidine group compared to the fentanyl group. Although both groups exhibited increased IL-6 and CRP levels postoperatively, without significant differences, further research is needed to validate the efficacy of dexmedetomidine in oral cancer pain management.

A multimodal approach to pain management was explored in Gunjan et al.’s study, involving three groups of patients undergoing oral cancer surgeries [108]. The multimodal approach in Group C, combining dexmedetomidine with nerve blocks and fentanyl, demonstrated superior pain control, reduced analgesic requirements, and higher patient satisfaction compared to other groups. The study highlights the potential benefits of this comprehensive pain management strategy for oral cancer.

Nair et al. investigated pain control in patients with head and neck cancers undergoing surgery [112]. The use of dexmedetomidine effectively attenuated the stress response, reduced bleeding, ensured smooth emergence, and enhanced tube tolerance in head and neck oncosurgeries. The findings suggested that Dexmed could be a valuable adjuvant in the management of postsurgical patients, offering stable hemodynamics and improved outcomes in major head and neck surgeries.

### 4.2. Efficacy of Adjuvant Therapies

Another interesting approach emerging from the literature is the use of gabapentin as an adjuvant in postoperative pain management. Chiu et al.’s study involved fifty patients divided into two groups, one treated with gabapentin and the other a control group. The preoperative administration of gabapentin demonstrated a significant reduction in postoperative pain, measured through decreased VAS scores and reduced morphine usage in the first 24 h post-surgery [104]. This suggests that gabapentin might have opioid-sparing benefits, reducing the need for morphine immediately after surgical procedures.

However, it is essential to note that Chiu et al.’s study acknowledges some limitations, such as its retrospective design, highlighting the need for additional research to establish the ideal dosage, cost-effectiveness, and long-term impacts on patient outcomes [104]. Despite these limitations, gabapentin emerges as a valuable adjuvant in oral cancer surgery.

In the context of postoperative pain management, the use of NSAIDs plays a relevant role. Puttaswamy et al.’s study compared aceclofenac and diclofenac in postoperative pain management for patients undergoing composite resection for oral cancer [106]. Both drugs effectively managed pain, with FLACC scores below three and a large decrease in VAS scores at 72 h post-surgery. The study indicates that aceclofenac could be considered a suitable alternative for pain management in oral cancer patients, particularly for those with a history of gastritis or peptic ulcers.

Another significant contribution to the literature comes from the research conducted by Yaguchi et al., focusing on the use of hydroxyethyl starch in oral cancer surgeries [105]. The study demonstrated that the administration of hydroxyethyl starch effectively maintained circulation without increasing intraoperative blood loss or adversely affecting renal function, even at lower doses. While acknowledging limitations, such as its retrospective nature and short follow-up period, the study suggests the potential effectiveness and safety of hydroxyethyl starch in oral cancer surgeries, providing valuable insights for further exploration.

### 4.3. Multimodal Approaches

Amiri et al.’s research delved into the effectiveness of preemptive analgesia (PAND) in radical neck dissection surgery for oral cancer patients [109]. The PAND regimen, comprising pregabalin, acetaminophen, naproxen, and dextromethorphan, significantly reduced postoperative pain and opioid analgesic requirements compared to the control group. The study suggested the need for further research to refine preemptive analgesic protocols for improved pain management in radical neck dissection surgery.

Another significant contribution comes from Singhal et al.’s investigation into postoperative pain control in oral cancer patients undergoing a commando operation with PMMF reconstruction [110]. The use of epidural morphine showed superior analgesia compared to intravenous morphine in oral cancer surgery with flap reconstruction. The study suggested that epidural morphine offers enhanced postoperative pain management, emphasizing its potential benefits over intravenous morphine.

Sjamsudin et al. aimed to investigate pain control in patients with stage 3 and 4 oral squamous cell carcinoma (OSCC) undergoing surgery as the primary treatment [113]. The study revealed a significant decrease in postoperative pain and anxiety and an increase in quality of life. The findings suggest the effectiveness of operative therapy in reducing pain and anxiety levels while enhancing the overall quality of life in OSCC patients.

### 4.4. Holistic Approaches

Jiang et al. analyzed postoperative oral cancer cases, comparing observation and control groups [111]. The study indicated significant differences in oral care effects, tumor marker expression, immune capacity, and patient satisfaction between the two groups. The combination of traditional Chinese medicine (TCM) anticancer decoction, chemotherapy, and nursing intervention demonstrated enhanced clinical efficacy, reduced adverse reactions, and improved patient outcomes. The study suggests that this comprehensive approach positively influences immune function, patient satisfaction, and survival quality in postoperative oral cancer cases.

In conclusion, the scientific literature thoroughly examines various perspectives and approaches to pain management in patients with oral cancer undergoing surgical interventions. From persistent opioid use to the assessment of adjuvant drugs, such as gabapentin, from multimodal approaches to preventive therapies, each study contributes to outlining a comprehensive framework of strategies aimed at improving patients’ quality of life and reducing postoperative pain. A personalized approach and the integration of different therapeutic modalities emerge as key elements in postoperative pain management in patients with oral cancer, offering valuable insights for clinical practice and guiding the direction of future studies in this field.

In recent years, the integration of genomics and precision medicine has revolutionized the landscape of oral cancer treatment. By elucidating the genetic alterations and molecular pathways underlying the development and progression of oral cancers, genomics has enabled the identification of novel therapeutic targets and personalized treatment strategies [114]. Precision medicine approaches, leveraging genomic profiling and molecular diagnostics, empower clinicians to tailor treatment regimens to the unique genetic profile of individual patients. This paradigm shift from a one-size-fits-all approach to a patient-centric model holds immense promise in optimizing treatment outcomes and minimizing adverse effects in oral cancer patients. Furthermore, the ongoing advancements in high-throughput sequencing technologies and computational algorithms continue to propel the field forward, fostering a deeper understanding of the molecular complexities of oral cancer and paving the way for innovative targeted therapies and precision oncology interventions [5,40].

## 5. Limitations

The review on postoperative pain control in oral cancer patients, while contributing valuable insights, is not without limitations that warrant careful consideration. One prominent constraint is the limited availability of studies addressing the specific nuances of managing pain in oral cancer cases. This scarcity, while not uncommon in emerging fields, underscores the need for further extensive research to establish a more robust knowledge base.

Another potential limitation is the inclusion criteria that exclusively favored English-language studies. This decision introduces a language bias, potentially excluding pertinent research conducted in languages other than English. The consequence is a potential oversight of valuable contributions from non-English literature, limiting the depth and inclusivity of the review.

The studies analyzed exhibit significant heterogeneity, as reflected in their diverse methodologies and sample sizes. Specifically, a subset of studies adopts a retrospective design with relatively small sample sizes, potentially limiting the generalizability and robustness of the findings. This heterogeneity introduces challenges in synthesizing results cohesively and may impede the ability to draw definitive conclusions regarding postoperative pain management in oral cancer patients. Consequently, caution is warranted when interpreting the outcomes, and further research incorporating larger, prospective studies is needed to validate and expand upon the findings obtained from these varied study designs. 

The diversity in methodologies and quality assessments across these studies poses a challenge in synthesizing findings cohesively, potentially impacting the overall strength of the evidence presented.

Publication bias, a phenomenon where journals tend to favor publishing studies with positive or statistically significant results, may influence the review’s conclusions. The exclusion of studies with negative or inconclusive findings may skew the overall interpretation of the literature towards more optimistic outcomes.

While the search strategy utilizing Boolean keywords is a strength, the possibility of missing relevant studies cannot be entirely ruled out. Variations in terminologies or indexing across databases might lead to inadvertent omissions, affecting the comprehensiveness of the search.

Despite the effort to mitigate bias through a three-reviewer selection process, there remains a potential for subjective judgment, introducing selection bias. Differences in interpretation among reviewers could impact the inclusivity and representativeness of the studies included in the review.

## 6. Conclusions

The review, despite these limitations, offers a thorough exploration of diverse approaches to postoperative pain management in oral cancer patients. It sheds light on persistent opioid use; explores the efficacy of adjuvant drugs, such as gabapentin; and advocates for a multimodal approach, emphasizing the multifaceted nature of pain control. A key takeaway from the review is the critical need for a personalized approach to pain management in oral cancer patients. Recognizing the individuality of pain perception and tailoring interventions accordingly emerges as a central theme, providing clinicians with valuable insights into optimizing patient care. The integration of different therapeutic modalities, encompassing both pharmacological and non-pharmacological strategies, emerges as a crucial element in achieving comprehensive postoperative pain management. This holistic approach aligns with the evolving paradigm in healthcare that acknowledges the complexity of pain experiences and addresses them through diverse means. While offering practical insights for clinical practice, the review also acts as a guiding compass for future research. The identified limitations underscore the necessity for more extensive, rigorous studies with standardized methodologies and diverse participant populations. This imperative push for further research aims to deepen our understanding of oral cancer pain management and refine evidence-based practices in the field.

## Figures and Tables

**Table 1:** Database search indicators.

Articles screening strategy	KEYWORDS: A: oral cancer; B: postoperative pain; C: postoperative analgesia.
	Boolean Indicators: A AND (B OR C)
	Timespan: 2003–2023
	Electronic databases: Pubmed; Scopus; WOS

**Table 2:** Descriptive summary of item selection.

Author (Year)	Study Design	Number of Patients	Average Age and Gender	Drugs Used	Outcomes
Cata et al., 2019 [103]	Retrospective study	A total of 362 patients who underwent curative-intent surgery for oral tongue cancers.	The median age of the patients in the study cohort is reported as 58 years, 58% of the patients are male.	The drugs used in the study primarily involve opioids, with information on preoperative opioid use, in-hospital postoperative opioid consumption, and opioid use between 90 and 365 days after surgery. Specific opioids mentioned include oral hydrocodone, codeine, fentanyl, hydromorphone, morphine, oxycodone, and tramadol, either individually or in combination.	Outcomes include preoperative pain intensity, opioid consumption before and after surgery, and recurrence status at six and twelve months post-surgery. The study also explores factors associated with persistent and chronic opioid use, such as preoperative pain, preoperative opioid use, and adjuvant therapy.
Chiu et al., 2014 [104]	Non-randomized open-label trial	The study includes fifty patients with tongue cancer.	The average age of the patients is reported as 64 years in the control group and 61 years in the gabapentin group. Gender distribution details are not explicitly mentioned	The primary drug used in the study is gabapentin, administered at a preoperative dose of 1200 mg. The study also mentions the use of morphine patient-controlled analgesia (PCA) for postoperative pain management.	The outcomes suggest that preoperative gabapentin administration significantly reduces postoperative pain, morphine usage, and side effects, presenting a potential opioid-sparing effect.
Yaguchi et al., 2023 [105]	Case-control study	96 patients who underwent oral cancer surgery	Not Specified	Patients in the H130 group were administered 6% HES130/0.4, while those in the control group received extracellular fluids such as Ringer’s acetate or lactate solution.	The study demonstrated that 6% HES130/0.4 effectively maintained circulation without increasing intraoperative blood loss or adversely affecting renal function.
Puttaswamy et al., 2023 [106]	Prospective study	76 patients undergoing composite resection for oral cancer.	Patients aged between 25 to 70 years; 15 males and 61 females.	Entanyl 25 mcg transdermal patch for all patients; Group A received aceclofenac 150 mg, and Group D received diclofenac sodium 75 mg intramuscularly.	Both drugs effectively managed pain, with a significant reduction in VAS scores at 72 h. Diclofenac demonstrated slightly better pain control. Adverse effects, primarily nausea and epigastric discomfort, were more common with diclofenac.
Gupta et al., 2018 [107]	Prospective comparative study.	60 patients, with 30 in each group (Group Ist and Group IInd)	Patients of ASA grade I or II, aged 18 to 70 years, included in the study. Specific details on average age and gender distribution are not provided.	Group Ist: Paracetamol (PCM) 10 mg/kg body weight, fentanyl 2 μg/kg body weight, propofol 2 mg/kg body weight, succinylcholine 2 mg/kg body weight, and vecuronium. Maintenance with oxygen, N_2_O, Isoflurane, and continued fentanyl. Group IInd: PCM 10 mg/kg, dexmedetomidine 0.5 μg/kg, propofol 2 mg/kg, succinylcholine 2 mg/kg, and vecuronium. Maintenance with oxygen, N_2_O, isoflurane, and continued dexmedetomidine.	Dexmedetomidine demonstrated better hemodynamic stability, reduced postoperative pain, and lower analgesic requirements compared to fentanyl in oral cancer surgery patients. Adverse effects were generally manageable in both groups. Further studies with larger sample sizes may provide additional insights.
Gunjan et al., 2016 [108]	Randomized clinical trial	30 patients in each of the three groups, totaling 90 participants.	The mean age of patients in Group A, Group B, and Group C were 50.53 ± 12.45, 44.67 ± 12.09, and 49.77 ± 13.14 years, respectively. The gender distribution was not explicitly mentioned.	Group A: Fentanyl 1 μg/kg. Group B: Fentanyl 1 μg/kg + bupivacaine local infiltration. Group C: Fentanyl 1 μg/kg + bupivacaine local infiltration	The outcomes were compared among the three groups, emphasizing the efficacy of the multimodal approach in Group C for improved pain control, reduced analgesic requirements, and higher patient satisfaction.
Amiri et al., 2016 [109]	Rrandomized clinical trial.	80 patients	In the PAND group, the mean age was 49.58 ± 13.96 years, with 21 females and 19 males. The control group had a mean age of 49.81 ± 14.59 years, with 18 females and 22 males.	Patients in the PAND group received 2.5 mg/kg pregabalin, 15 mg/kg acetaminophen, 7 mg/kg naproxen, and 0.3 mg/kg dextromethorphan, administered orally one hour before surgery.	The study reported a statistically significant reduction in postoperative pain in the PAND group, with a 54% lower opioid analgesic requirement compared to the control group.
Singhal et al., 2006 [110]	Prospective randomized trial	60 patients	The patients selected for the study ranged in age from 25 to 60 years.	Patients were randomized to receive either epidural morphine analgesia or intravenous morphine during the post-operative period.	Epidural morphine offers better pain control than intravenous morphine after oral cancer surgery
Jiang et al., 2022 [111]	Randomized clinical trial.	84 postoperative oral cancer cases	The average age included in the study was 44–71 years.	The drugs used in the study included a basic chemotherapy regimen for the control group, consisting of vincristine, pingyangmycin, and dexamethasone. The observation group received a Traditional Chinese Medicine (TCM) anticancer decoction.	The results indicated significant differences between the observation and control groups in terms of oral care effects, tumor marker expression, immune capacity, patient satisfaction, and clinical efficacy, suggesting that the comprehensive approach involving TCM anticancer decoction, chemotherapy, and nursing intervention positively influenced patient outcomes.
Nair et al., 2022 [112]	Randomized clinical trial.	150 patients	The age range of the patients was between 18 and 65 years. Gender distribution was not explicitly mentioned.	The drugs used in the study included dexmedetomidine (Dexmed) administered to group D, and saline administered to group S.	The study concluded that dexmedetomidine, when given as an IV bolus followed by an infusion at 0.4 mcg/kg/h, effectively attenuated the stress response during intubation and throughout the surgical duration. It also resulted in less bleeding.
Sjamsudin et al., 2018 [113]	Randomized clinical trial.	The study recruited 21 patients with stage 3 or stage 4 OSCC.	The age range of the recruited patients was 27 to 74 years old, with a mean age of 48.05 years. The gender distribution included 10 males and 11 females.	The text mentions the involvement of analgesics in postoperative pain management, but specific drug names are not provided.	The study concludes that there was a significant decrease in oral cancer pain level and anxiety level in OSCC patients after operative procedures, regardless of the next course of treatment. Additionally, participants’ quality of life increased significantly after the operative procedure, indicating the positive impact of operative therapy on pain control and overall well-being in OSCC patients.

## Data Availability

No new data were created or analyzed in this study. Data sharing is not applicable to this article.

## Abbreviations

EORTC QOL	European Organization for Research and Treatment of Cancer Quality of Life Questionnaires
FLACC	Face, Legs, Activity, Cry, Consolability
HADS	Hospital Anxiety and Depression Scale
NSAID	Non-steroidal anti-inflammatory drug
UPAT	Universal Pain Assessment Tool
OSCC	Oral squamous cell carcinoma
UCSF-OCPQ	University of California San Francisco Oral Cancer Pain Questionnaire
PAND	Preemptive analgesia
PCA	Patient-controlled analgesia
PMMF	Pectoralis major myocutaneous flap
TCM	Traditional Chinese Medicine
VAS	Visual analog scale
